# Formulation of Ocular In Situ Gels with Lithuanian Royal Jelly and Their Biopharmaceutical Evaluation In Vitro

**DOI:** 10.3390/molecules26123552

**Published:** 2021-06-10

**Authors:** Kristina Perminaite, Mindaugas Marksa, Monika Stančiauskaitė, Tadas Juknius, Aidas Grigonis, Kristina Ramanauskiene

**Affiliations:** 1 Department of Clinical Pharmacy, Faculty of Pharmacy, Lithuanian University of Health Sciences, Sukileliai Ave. 13, 50162 Kaunas, Lithuania; monika.stanciauskaite@lsmuni.lt (M.S.); Kristina.ramanauskiene@lsmuni.lt (K.R.); 2 Faculty of Pharmacy, Institute of Pharmaceutical Technologies, Lithuanian University of Health Sciences, Sukileliai Ave. 13, 50162 Kaunas, Lithuania; 3 Department of Analytical and Toxicological Chemistry, Lithuanian University of Health Sciences, Sukileliai Ave. 13, 50162 Kaunas, Lithuania; mindaugas.marksa@lsmu.lt; 4 Faculty of Veterinary Medicine, Institute of Microbiology and Virology, Lithuanian University of Health Sciences, Tilzes Str. 18, 47181 Kaunas, Lithuania; tadas.juknius@gmail.com; 5 Dr. L. Kriaučeliūnas Small Animal Clinic, Veterinary Academy, Lithuanian University of Health Sciences, Tilzes Str. 18, 47181 Kaunas, Lithuania; aidas.grigonis@lsmuni.lt

**Keywords:** royal jelly, in situ gels, poloxamer, ocular, Statens Seruminstitut Rabbut Corneal cells, 10-hydroxy-2-decenoic acid, antioxidant, antibacterial

## Abstract

Royal jelly is a natural substance produced by worker bees that possesses a variety of biological activities, including antioxidant, anti-inflammatory, antibacterial, and protective. Although fresh royal jelly is kept at low temperatures, to increase its stability, it needs to be incorporated into pharmaceutical formulations, such as in situ gels. The aim of this study was to formulate in situ ocular gels containing Lithuanian royal jelly for topical corneal use in order to increase the retention time of the formulation on the ocular surface and bioavailability. Gels were evaluated for physicochemical characteristics (pH, rheological properties, refractive index) and in vitro drug release measuring the amount of 10-hydroxy-2-decenoic acid (10-HDA). An ocular irritation test and cell viability tests were performed using the SIRC (Statens Seruminstitut Rabbit Cornea) cell culture line. Results indicated that all the in situ gels were within an acceptable pH and refractive index range close to corneal properties. Rheology studies have shown that the gelation temperature varies between 25 and 32 °C, depending on the amount of poloxamers. The release studies have shown that the release of 10-HDA from in situ gels is more sustained than royal jelly suspension. All gel formulations were non-irritant according to the short-time exposure test (STE) using the SIRC cell culture line, and long-term cell viability studies indicated that the formulations used in small concentrations did not induce cell death. Prepared in situ gels containing royal jelly have potential for ocular drug delivery, and they may improve the bioavailability, stability of royal jelly, and formation of non-irritant ocular formulations.

## 1. Introduction

Royal jelly (RJ) is a yellowish white, creamy complex bee product produced by mandibular glands of worker bees. [1,2]. Pharmacological data published have proven that RJ possesses various biological activities, including antioxidant, anti-inflammatory, antimicrobial, antidiabetic, anti-cancer, and many others [1,2,3,4,5,6]. The chemical composition of RJ is very rich, consisting of proteins, amino acids, sugars, polyphenols, phenolic compounds, as well as lipids [7,8]. The biological activity of RJ is directly attributable to the bioactive compounds in the composition.

One of the most biologically active compounds of RJ is *trans*-10-hydroxy-2-decenoic acid (10-HDA), which is often referred to as queen bee acid and is the predominant fatty acid constituent [9]. This fatty acid is naturally found only in RJ, and it can be used as a quality parameter for the authenticity of RJ samples [10,11].

10-HDA possesses various biological activities, including anti-inflammatory, antimicrobial, and antioxidant [12,13]. Although the data on ocular use of RJ are limited, the scientific data published indicated the positive impact of RJ and 10-HDA supplementation for dry eye syndrome [14], and RJ was proven to heal the corneal alkali burns when used topically [15]. Even though RJ and 10-HDA possess various biological activities, they are normally stored in freezer (−18 °C temperature), and the physical appearance as well as biological activity decreases while maintaining room temperature. In order to achieve the stabilization and protection of biological activities, it is appropriate to incorporate them into pharmaceutical forms, such as in situ forming gel.

In situ gels are promising ocular drug delivery systems based on the in situ gel formation, which ensures longer precorneal residence time and improved ocular bioavailability, comparing to conventional topical eye drops [16,17]. The in situ gels are polymeric solutions that are liquid in room temperature, and they form gel only when applied to the conjunctival sac, which forms a viscoelastic gel, overcoming the barrier of the nasolacrimal drainage and washing out by tear secretion [18]. Polymers that are used for semisolid preparations are poloxamers, which are synthetic polymers that exhibit thermoresponsive behavior, with an easy tunable gelation temperature [19]. Poloxamer 407 (P407) and Poloxamer 188 (P188) are polymers that are widely used in ocular drug formulations because of their good solubility in water, clarity, and shear thinning behavior of their solutions, and safety to ocular tissues [20,21]. The aim of this study is to model ocular in situ gels with royal jelly and pure 10-HDA and evaluate their quality and biological activity in vitro.

## 2. Results and Discussion

### 2.1. Determination of 10-HDA in RJ Samples

In the first stage of this study, the total amount of 10-HDA in royal jelly samples was determined using the HPLC method. 10-HDA is specific to RJ and a stable compound, and it can be used as an authenticity parameter [10,11].

Three various series of Lithuanian RJ samples were taken for this study, which were collected in 2018 (RJ1), 2019 (RJ2), and 2020 (RJ3). The results are shown in Table 1.

The amount of 10-HDA determined in Lithuanian RJ samples varied between 2.58 and 3.63% (*w/w*), which shows that the amount of 10-HDA in various samples can vary significantly. The amount of 10-HDA in RJ samples depends on the place where it was collected, the time of the year, and many other environmental factors [22,23]. Even though the amount of 10-HDA determined in various samples of RJ may seem like a possible freshness parameter, the scientific data prove that the amount of 10-HDA does not depend on the freshness of RJ [23]. The studies have shown that in the freezer, the quality of RJ does not change for 2–3 years [24].

RJ samples with the highest amount of 10-HDA (RJ3) were used for further studies and in situ gel preparation.

### 2.2. Antioxidant Activity of RJ and 10-HDA

The next step in the study was to evaluate the antioxidant activity of Lithuanian RJ and 10-HDA. The results of the experiments are indicated in Table 2.

RJ is a complex bee product, and in order to properly evaluate the antioxidant activity, as for most of the substances of natural origin, it is valuable to perform multiple tests [25].

The results of antioxidant activity have shown that RJ possess higher antioxidant activity comparing to pure 10-HDA in all assays. A 5% RJ suspension possessed significantly higher (*p* < 0.05) antioxidant activity compared to 1% RJ suspension. The study showed that the highest in vitro antioxidant activity by all methods was for 5% RJ suspension in PBS (51.563% by ABTS, 48.277% by DPPH, and 45.473% by FRAP). In comparison, 1% RJ suspension in PBS possessed the antioxidant activity from 15.054 (FRAP) to 19.385% (ABTS). 10-HDA at all concentrations had the antioxidant activity lower than 10% measuring with ABTS and DPPH methods, and it was lower than 1% when measured using the FRAP method. Such unequal results could have been derived due to the applied methods limitations. The DPPH method is used mainly for hydrophobic antioxidants, and the FRAP method is limited to the compounds that are not based on hydrogen transference reactions [26,27].

RJ, in comparison to other bee products such as propolis and bee bread, exhibits lower antioxidant activity [28]. Current research indicates that the antioxidant activity of RJ can be attributed to the specific protein fraction MRJP 2 (Major royal jelly proteins) [29].

### 2.3. The Determination of Antimicrobial Activity of RJ and 10-HDA

The next stage of the study was to determine the antimicrobial activity of RJ and 10-HDA samples evaluating the effect on 5 different reference American Type Culture Collection (ATCC) strains. The results of this experiment are shown in Table 3.

RJ suppressed best the growth of *E. coli* (15.27 mm disc diameter), *P. aeruginosa* (13.667 mm disc diameter), and *C. albicans* (13.33 mm disc diameter). RJ had significantly lower impact (*p* < 0.05) on the *S. aureus* strain (11.333 mm disc diameter). Both RJ and 10-HDA had the lowest antibacterial activity on *B. cereus* (8.333 and 3.331 mm disc diameter, respectively). 10-HDA shower the highest antimicrobial activity against *C. albicans* (25.164 mm disc diameter), and it showed activity against *P. aeruginosa, E. coli,* and *S. aureus* bacterial strains (14.264, 18.334, and 15.141 mm disc diameter, respectively).

Bee products, including RJ, have been proven to exhibit antimicrobial activity, which can be attributed to the biologically active compounds, such as 10-HDA [30,31]. The bacterial and yeast strains used in this study are directly related to ocular infections [32,33,34,35,36].

The results of this study indicate that both pure 10-HDA and RJ are potential candidates for the eye drops with antimicrobial activity.

### 2.4. The Physiochemical Parameters of Prepared In Situ Gels

In this study, in order to incorporate the RJ and 10-HDA and to perform the studies on cell-culture models, in situ gels were prepared. The compositions of in situ gel formulations are indicated in Table 4.

**Figure 1:**
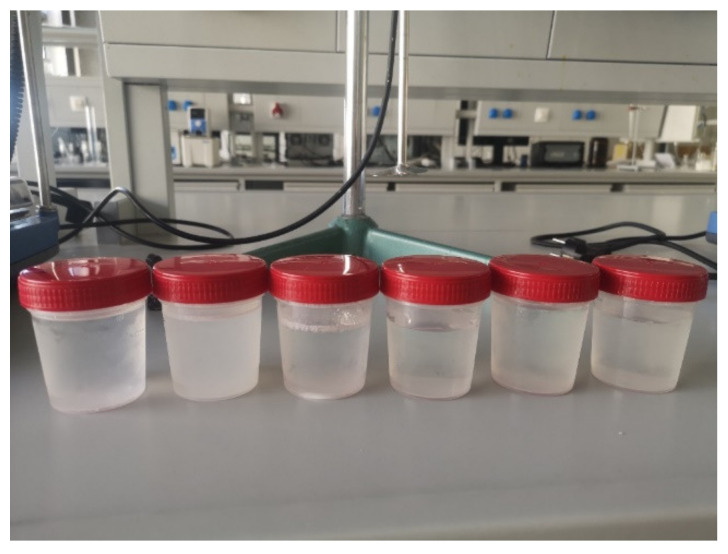
The appearance of the in situ gels prepared. Gel formulations N1–N6 (left to right).

The in situ gel formulations prepared were clear, transparent, without any visible particles, and liquid in room temperature (Figure 1).

After the preparation of in situ gels, the quality determination was performed. The results of the determination of physicochemical parameters of prepared in situ gels are indicated in Table 5.

All the gels prepared were clear and transparent liquids at 4 °C (Figure 1, Table 5). The refractive index of the in situ gel formulations at the physical temperature (37 °C) varied between 1.322 (N9) and 1.432 (N8), which means within an acceptable range for ocular formulations. The refractive index of the cornea is around 1.38 [37]. It is recommended that the refractive index of ocular formulations would not be higher than 1.476 [38]. The pH of the in situ gels prepared varied between 4.98 and 5.96. The pH of the ocular formulations is directly related to its tolerability while applied to the ocular surface. For the maximum ocular comfort, opthalmic preparations should be close to neutral (7.2) [39], yet the studies performed previously indicate that the pH of ocular formulations can vary between 3.5 and 8.5 [40].

The gelation temperature ($T_{Sol-Gel}$) is one of the main factors for the evaluation of quality of in situ gels. The in situ gels prepared were evaluated rheologically for the gelation temperature, which indicates the point where the solution undergoes the gelation process and changes to semisolid phase.

Thermosensitive gels, prepared using poloxamers, are liquid at cold temperatures, and while the temperature increases, gel formation starts. For the preparation of the in situ gels, poloxamers 407 and 188 were used, which have been previously proven safe for the ocular formulations, and they can be sterilized by autoclaving [41,42]. As reported previously, the gelation temperature of P407 solutions is quite low (22–26 °C) when used in safe concentrations, and there is a relatively high gelation temperature while using P188 alone (often 40 °C and more) [42]. However, while using these two poloxamers in mixture, formulations with gelation temperature closer to physiological can be obtained [43]. The most suitable $T_{Sol-Gel}$ for ocular formulations is well below the temperature at the surface of the eye (35 ° C) but higher than room temperature (25° C) [44]. Prepared in situ gels with RJ had the $T_{Sol-Gel}$ 24.5–30.5 °C, while gels with pure 10-HDA had the $T_{Sol-Gel}$ 25.5–30 with the decrease of gelation temperature, while the concentration of poloxamers increases. There was no statistical difference between gels with RJ and with 10-HDA (*p* > 0.05). In order to ensure the quality of prepared gels, it is advised to keep them in the refrigerator (4 °C) and keep them at room temperature for several minutes for the comfort during use and to avoid gelation. The $T_{Sol-Gel}$ of the prepared gels has corresponded to the values specified for opthalmic formulations [45].

Another important step in formulating thermosensitive ocular in situ gels is the determination of viscosity at various temperatures. The dynamic viscosity at 4 °C varied between 17.1 and 43.2 mPas; at 22 °C, it was 18.1–82.3 mPas. The dynamic viscosity of in situ gels was the highest, as all of the formulations at the temperature of 35 °C have already undergone the gelation point, and it was 44.1 mPas when the concentrations of poloxamers were lowest (N10) and up to more than 10 Pas when the concentration of poloxamers was the highest (18%P407/10% P188 solution). The results have shown that when the temperature increases, the viscosity increases significantly (*p* < 0.05). In addition, the amount of poloxamers affected the viscosity of the formulated in situ gels. The increase of viscosity allows the formulations to avoid the tear drainage effect and enhances the bioavailability of the formulations. However, in the semisolid gel form, our in situ gel formulations have possessed high viscosity, which could cause blurred vision for the short period of time, so the formulation would be recommended to be administered at night time [46]. The scientific data report that the major part of active substances applied to the corneal surface are washed out due to the increased tear secretion minutes after the application [47], and the in situ gels can overcome this barrier and increase the bioavailability of the active substances introduced to the eye. Ocular in situ gel formulations, according to the literature, should have viscosity of 5–1000 mPas before gelling, and after gel formation, the viscosity should be from 50 to 50,000 mPas [48].

### 2.5. The Antioxidant Activity of In Situ Gel Formulations Evaluated by DPPH Method

**Figure 2:**
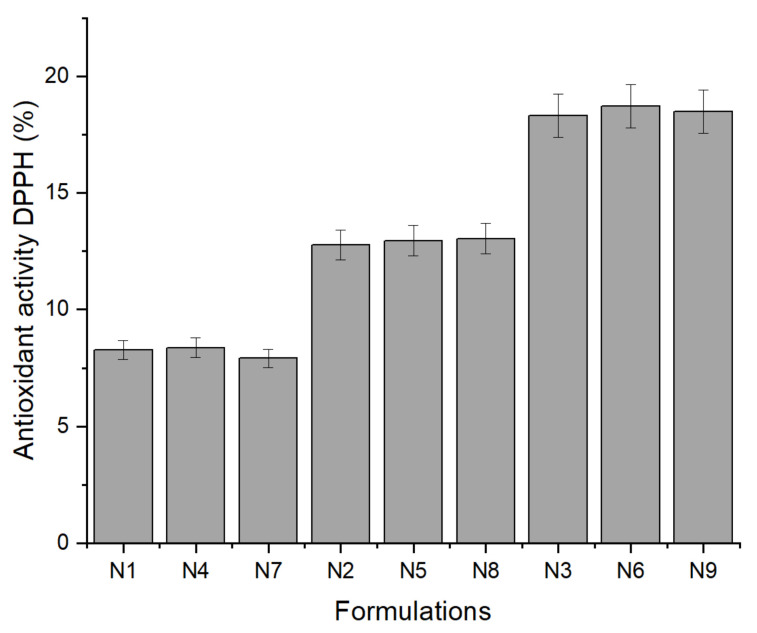
The antioxidant activity of in situ gel formulations measured with the DPPH method. Data represent mean standard deviation (SD), *n* = 3.

After evaluating the physicochemical parameters of prepared in situ gels, their antioxidant activity was evaluated by the DPPH method. The results are shown in Figure 2.

The results have shown that there was no difference between the formulations with the same amount of RJ, and empty gel formulations did not possess the antioxidant activity. In the formulations with 0.5% of RJ (N1, N4, and N7), the antioxidant activity was the lowest. In the formulations with 0.75% of RJ (N2, N5, and N8), the antioxidant activity was significantly higher (*p* < 0.05). In the formulation with 1% of RJ, the antioxidant activity was the highest, and it did not significantly differ from 1% RJ suspension, which was evaluated prior to the preparation of the in situ gels (*p* > 0.05). The formulations with pure 10-HDA (N10, N11, and N12) did not possess the antioxidant activity, the reason being that pure 10-HDA did not show high antioxidant activity when measured prior the production of the in situ gel formulations, and the antioxidant activity of royal jelly is attributed to other biologically active compounds [29].

### 2.6. The In Vitro Release Study of 10-HDA from In Situ Gels

Prior to the in vitro release of 10-HDA from in situ gel formulations, the total amount of 10-HDA in all formulations was determined using the HPLC method. The results are shown in Table 6.

The results indicate that the amount of 10-HDA determined by HPLC depended mainly on the amount of RJ or pure 10-HDA added to the formulation. The highest amount of 10-HDA was detected in in situ gel formulation N12, where the 0.002% (*w/v*) of pure 10-HDA was added.

**Figure 3:**
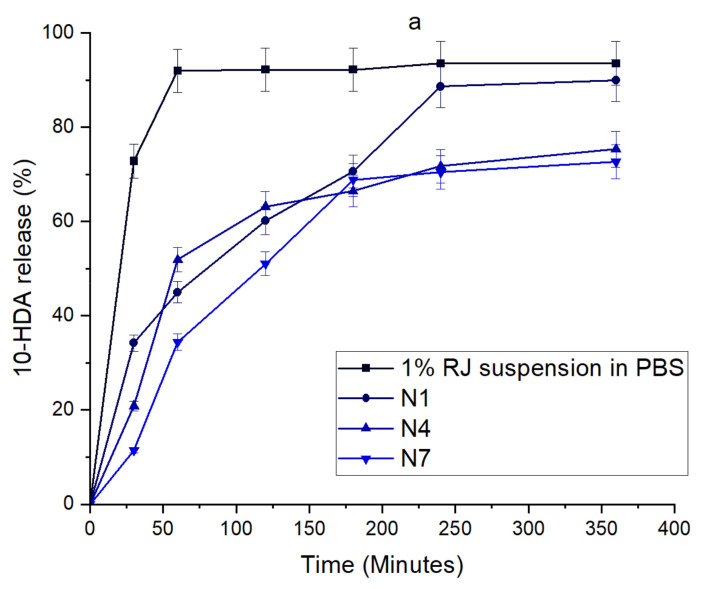

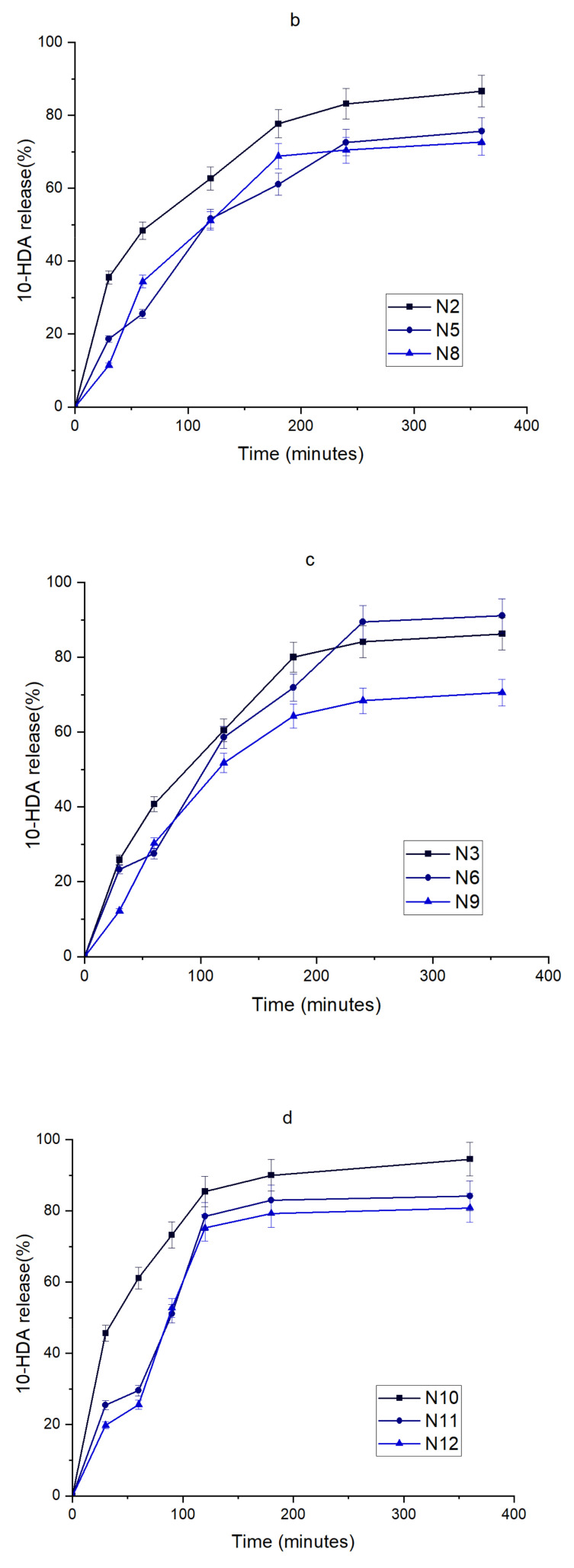
The release of 10-HDA from in situ gels and 1% RJ suspension. 1% RJ suspension and gel formulations N1–N3 (**a**), gels N4–N6 (**b**), gels N7–N9 (**c**), and gels N10–12 (**d**). Data represent mean standard deviation (SD), *n* = 3.

The in vitro release of 10-HDA from all in situ gels is shown in Figure 3.

The formulation with 1% RJ suspension was used as the control in order to evaluate the modified release profiles of in situ gels. The maximum amount (93.376%) of 10-HDA from 1% RJ suspension was released after 60 min. The maximum amount of 10-HDA from all in situ gel formulations was released after 6 h. There was no significant difference between the percentage of 10-HDA released from the formulations N1–N3 (*p* > 0.05). The amount released from the in situ gel N6 was statistically significantly higher (91.114%) in comparison with gels N4 and N5 (75.358 and 75.647%, respectively) (*p* < 0.05). There was no statistically significant difference between the amounts of 10-HDA released from the formulations N7–N9 (*p* > 0.05). From the formulations with pure 10-HDA N10, N11, and N12, the amount of 10-HDA released was 80.862, 84.211, and 94.535%, respectively. The in vitro release experiments have shown that when the amount of P407 in the in situ gel increased, the total amount of 10-HDA released after 6 h from the formulations was significantly lower (*p* < 0.05). In situ gels with a higher amount of P407 also possessed higher viscosity. The highest amount of 10-HDA was released when the amount of P407 in the formulations was the lowest (*p* < 0.05).

The in situ gel formulations prepared are suitable for ocular formulations as they increase the ocular retention time comparing to conventional eye drops, hence increasing the bioavailability [49]. When applied to the ocular surface, they undergo the transition to gel form and form the film on the surface, which is similar to a temporary lens [50]; thus, the retention time increases until the gel disintegrates due to the blinking and ocular drainage [50,51]. The in vitro tests have shown that the in situ gels have prolonged the release of 10-HDA from formulations in comparison to 1% RJ suspension.

### 2.7. The Stability Test of In Situ Gels

The stability of the prepared in situ gels was evaluated after 2 weeks. The in situ gels were evaluated measuring pH and the amount of 10-HDA by the HPLC method. The results are shown in Table 7.

The results shown in Table 6 indicate that after one and two weeks, there was no significant change of the amount of 10-HDA in all of the in situ gel formulations (*p* > 0.05). The pH of the formulations measured after one week did not decrease significantly (*p* > 0.05). Meanwhile, measuring the pH of the formulations after two weeks, there was a statistically significant change of pH in the formulations N4–N6, which had the gel base of 15% P407/13% of P188 solution. The pH decreased significantly in the in situ gel formulations N9 and N10, which had the gel base of 18% P407/10% of P188 solution. The reason for that could be the disintegration of RJ proteins. Regarding the in situ gel formulations containing pure 10-HDA, the pH has decreased significantly in the formulations N10 and N11 after two weeks. It is suggested that the change of pH is conditioned by the disintegration process, which usually starts when the temperature increases as the pure materials are kept in the freezer (−18 °C). The pH of pure RJ is around 4 [24]. When kept at room temperature, the disintegration process of RJ starts after 20 h [24]. In order to slow down the disintegration process, it is recommended to use the freeze-dried RJ, or the pure components of the RJ, yet the aim of this study was to incorporate fresh royal jelly into the in situ gel formulations. In order to keep the pH of the formulations at the means closer to neutral, and to decrease the risk of irritation, it is recommended to use the buffer systems [52].

### 2.8. SIRC Cell Culture Viability Tests

Corneal damage, using irritating and inflammation-inducing products, can cause discomfort and in severe cases ocular tissue corrosion, which can lead to temporary or permanent blindness. In order to properly evaluate ocular formulations, eye irritation potential and toxicity using corneal epithelial cells must be tested in order to ensure the safety of the formulations prepared [53]. In this study, the experiments with SIRC-cultured cells were performed after 24 h, and a short-term exposure (STE) test was performed to determine the eye irritation potential of the prepared in situ gels.

#### 2.8.1. MTT

**Figure 4:**
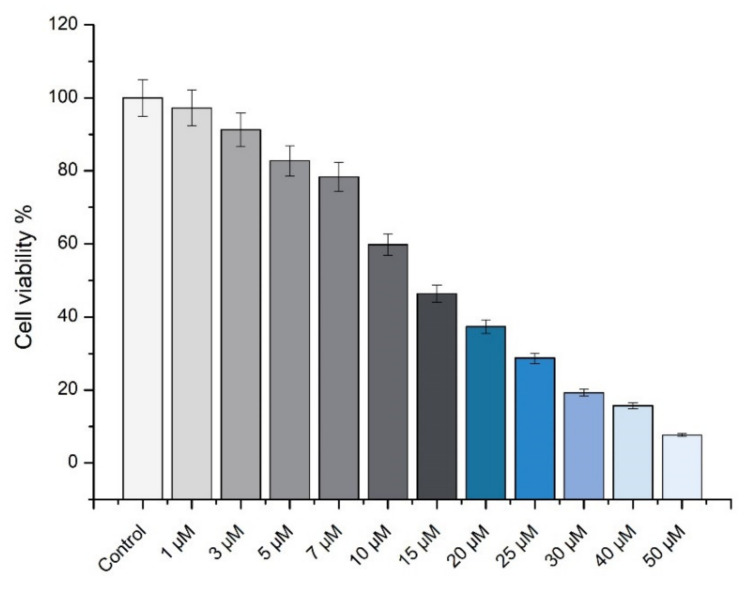
The SIRC cell viability after 24 h exposure with pure 10-HDA solutions. Data represent mean standard deviation (SD), *n* = 3.

The MTT test was performed after 24 h using pure 10-HDA in order to evaluate the cell toxicity and the safe concentrations for use for ocular formulations. The SIRC cell viability results using pure 10-HDA are shown in Figure 4.

The concentrations of 1–50 µM of 10-HDA were used for the cell toxicity experiment. The IC50 value was 12.8 µM. Concentrations of 1–3 µM did not cause a significant decrease of cell viability (*p* > 0.05). At concentrations of 5–50 µM, the cell viability decreased from 82.76% to 7.67%. The concentrations used in the in situ gels were safe (0.001–0.002% *w/v*) and did not induce cell death.

**Figure 5:**
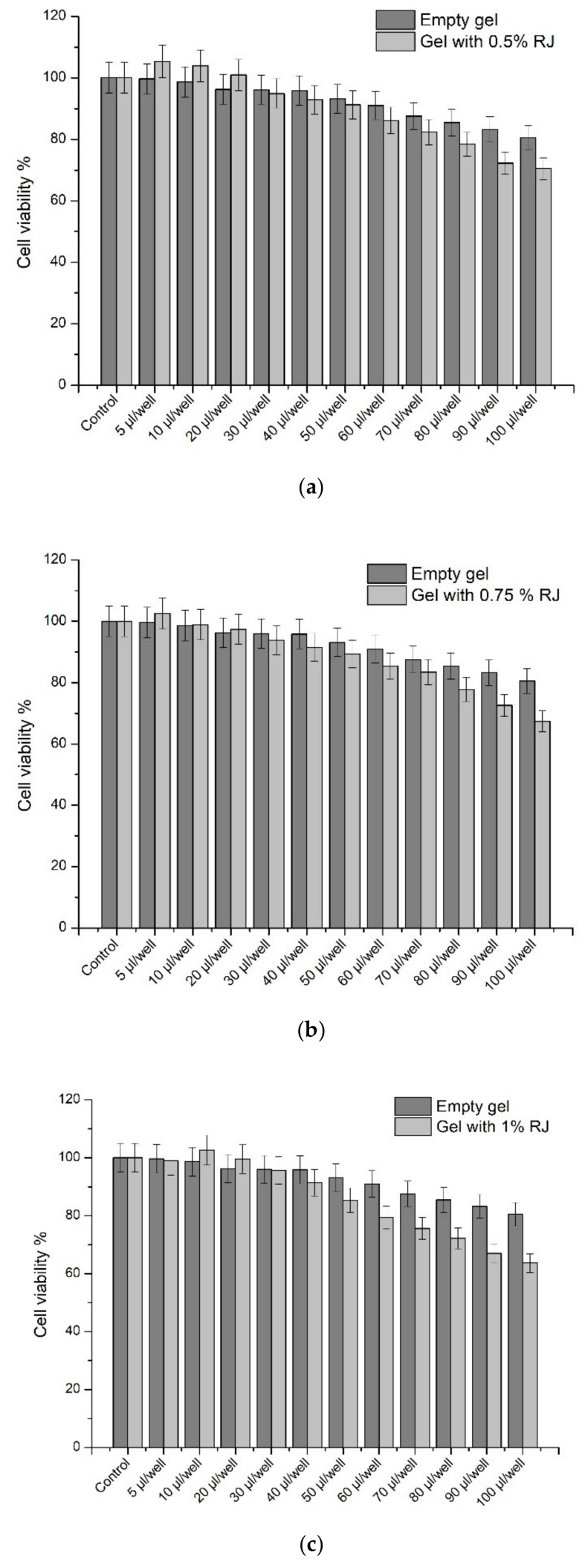

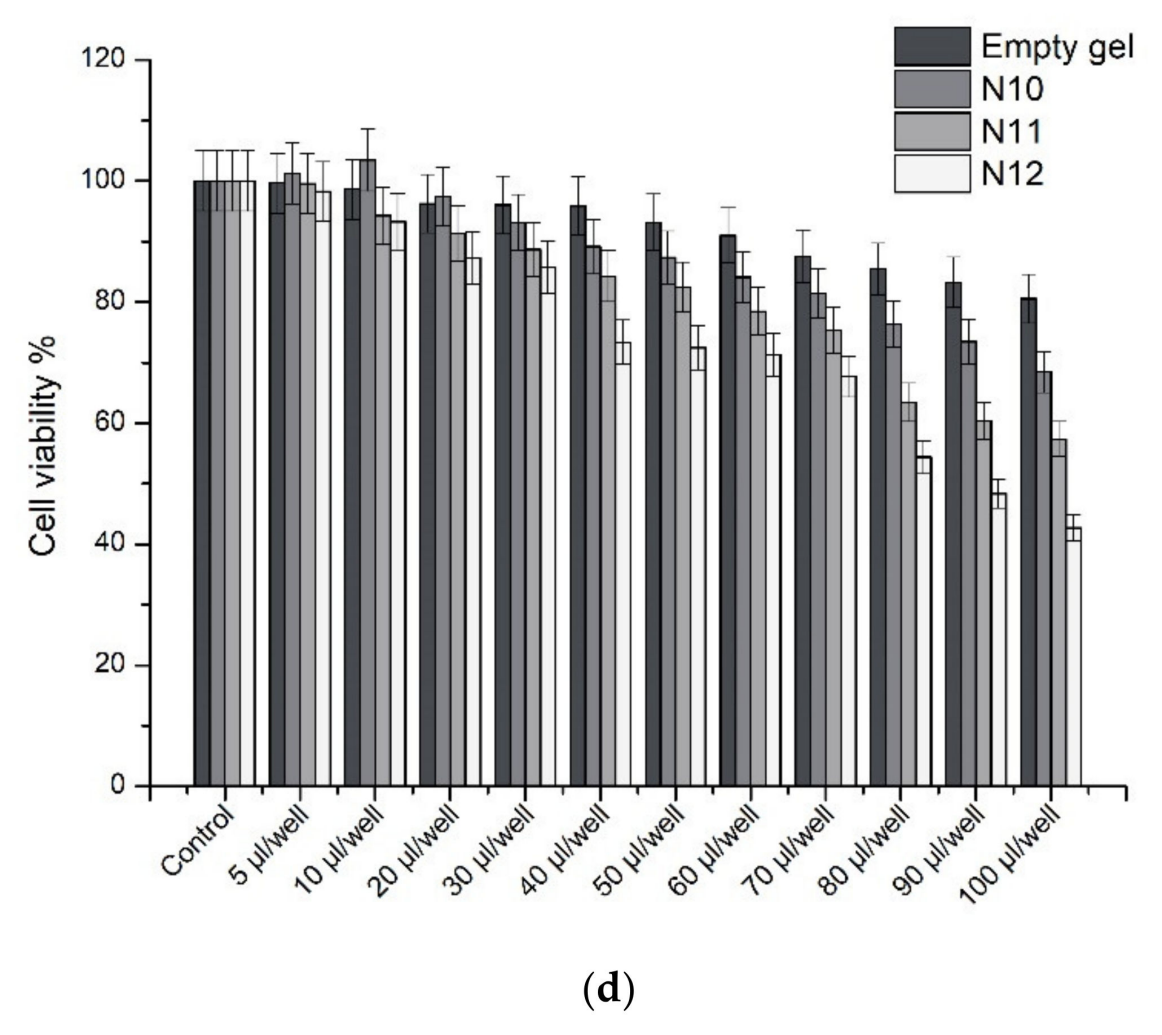
The cell viability results of all in situ formulations on the SIRC cell culture line after 24 h. (**a**) gel with 0.5% RJ, (**b**) gel with 0.75% RJ, (**c**) gel with 1% RJ, and (**d**) N10, N11, N12. An empty gel formulation was used in order to evaluate the impact of the excipients for the cell viability. Data represent mean standard deviation (SD), *n* = 5.

After the evaluation of cell viability after 24 h with pure 10-HDA, all of the in situ gels were exposed to cells, and their viability was assessed after 24 h of exposure. The results are shown in Figure 5.

The results of cell viability after 24 h incubation and exposure to the in situ gels have shown that the cell viability depends on the concentration of RJ and 10-HDA in the formulation. In situ gels N1, N4, and N7 had 0.5% (*w/v*) of RJ in their composition, and they have not impacted cell viability when applied 5-60 µL/well (*p* > 0.05). Using the amounts of 70–100 µL, the cell viability decreased significantly, from 86.14 to 70.48% (*p* < 0.05). In situ gels N2, N5, and N8, with 0.75% (*w/v*) of RJ in the composition, have not significantly changed cell viability at the amounts 5–50 µL/well (*p* > 0.05). Using 60–100 µL/well, cell viability decreased from 85.37 to 67.38% (*p* < 0.05).

In situ gels N3, N6, and N9, with 1% (*w/v*) of RJ in the composition, have not significantly changed cell viability at the amounts 5–40 µL/well (*p* > 0.05). Using 50–100 µL/well, cell viability decreased from 85.36 to 63.68% (*p* < 0.05). In situ gel N10 with 0.001% (*w/v*) of 10-HDA in the composition did not significantly change cell viability at the amounts 5–30 µL/well (*p* > 0.05). Using 40–100 µL/well, cell viability decreased from 89.14 to 68.46% (*p* < 0.05). In situ gel N11 with 0.0015% (*w/v*) of 10-HDA did not significantly change cell viability at the amounts 5–20 µL/well (*p* > 0.05). Using 30–100 µL/well, cell viability decreased from 88.68 to 57.38% (*p* < 0.05). The in situ gel N12 with 0.002% (*w/v*) of 10-HDA in the composition did not have a significant effect on cell viability when we used 5–10 µL/well (*p* > 0.05). Using 20–100 µL/well, the cell viability decreased from 87.28 to 42.69% (*p* < 0.05). IC50 was 84 µL/well.

The empty gel did not have a significant effect on the decrease of cell viability when using the amounts 5–60 µL/well (*p* > 0.05), and using the amounts 70–100 µL/well decreased the cell viability from 91 to 80.53% (*p* < 0.05).

The results of this long-term cell viability experiment have shown that all of the in situ gel formulations used in small amounts for 24 h did not induce cell death; thus, they can be safely used as non-irritant formulations. SIRC cell viability is defined as the percentage of living cells evaluated by their ability to metabolize MTT dye. Increased cell viability, while in comparison to the control (100%), means that the components and formulation have increased cell proliferation and it is safe to say that the formulations are safe and non-toxic, whereas a decrease would mean that the components of the formulation and their concentration may be potentially toxic to the cell cultures [54,55,56]. The results have shown that when using formulations containing 0.5% of RJ in small amounts, the cell viability increased, and the gel with the highest amount of pure 10-HDA in higher amounts decreased cell viability significantly. However, the formulations for the cell viability studies were applied directly to the cells, and while administering the final product, the concentration in which the active compounds reach the cell layers after bypassing the tear drainage decreases significantly. The cell viability experiment after 24 h was performed in order to ensure there was no long-term toxic effect with all of the formulations.

#### 2.8.2. Short-Term Exposure (STE) Using SIRC Cell Cultures

**Figure 6:**
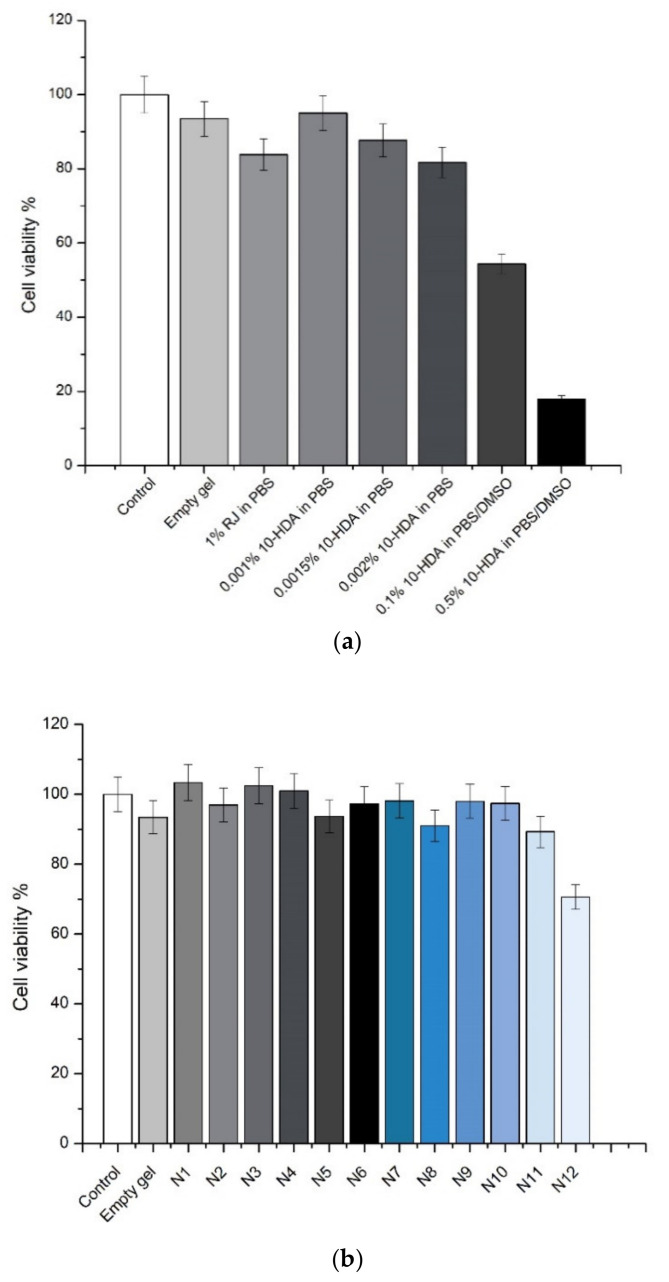
The STE test results after 30 min of exposure to RJ and 10-HDA solutions (**a**) and all in situ gels (**b**). Data represent mean standard deviation (SD), *n* = 5.

The STE test results using various RJ and 10-HDA solutions and in situ gels are shown in Figure 6.

The SIRC cell culture line is one of the most widely used eye irritation tests avoiding the excess use of animals. Prior to the testing, water-soluble substances can be solubilized in water and PBS, and water-insoluble substances can be solubilized in mineral oil or in the mixture of dimethylsulfoxide/PBS. 10-HDA is a fatty acid, which is partly soluble in PBS, and a higher amount needs to be solubilized using a small amount of ethanol or dimethylsulfoxide, because both of these solvents are toxic to the cells. The results of the test are being interpreted according to the cell viability. If the cell viability is 70% or more, the substance or formulation is accepted as non-irritant, and if cell viability is lower than 70%, the substance or formulation is accepted as irritant [54].

The results have shown that the 10-HDA solutions with concentrations higher than 0.002% (*w/v*) decreased cell viability significantly and are irritant (0.1% 10-HDA: 54.38%, 0.5% 10-HDA: 17.95%). All in situ gel formulations did not induce cell viability decrease by more than up to 90.9%, and in situ gel N12, with 0.002% 10-HDA, did decrease cell viability by up to 70.9% (*p* < 0.05); thus, the highest safe concentration of pure 10-HDA in ocular formulation could be 0.002%.

The STE test nowadays is used in place of the Draize test with albino rabbits, which is one of the most common ocular irritation tests, in the first stages of modelling ocular formulations [55]. While performing the STE, the cells are exposed to the formulations or solutions tested for 5 min, because usually, that is the maximum contact time with the ocular surface. Although, for the in situ formulations, the test time could be increased to 30 min, because the gel stays longer on the eye surface [56].

## 3. Materials and Methods

### 3.1. Materials

Lithuanian royal jelly (RJ) was purchased from “Bičių korys“ (Kaunas, Lithuania). Analytical grade 10-hydroxy-2-decenoic acid (10-HDA) was purchased from Sigma-Aldrich Chemie GmbH (Steinheim, Germany). Pluronic^®^ F-127 (P407) and benzalkonium chloride were supplied by Sigma-Aldrich Chemie GmbH (Steinheim, Germany). Sodium acetate, glacial acetic acid, glycerin, sodium bicarbonate, sodium chloride, and calcium chloride dihydrate were used for ocular buffer solution and purchased from Sigma Aldrich Chemie GmbH (Steinheim, Germany). Sterile water for injections was purchased from Sanitas (Kaunas, Lithuania).

The SIRC cell culture line was purchased from ATCC. Trypsin-EDTA, Eagle‘s Minimum essential medium, penicillin/streptomycin solution, fetal bovine serum (FBS), Pluronic^®^ F-68 (P188) sterile 10% solution, and 3-(4,5-dimethylthiazol-2-yl)-2,5-diphenyltetrazolium bromide (MTT) were obtained from Life technologies (Thermo Fisher Scientific, Waltham, MA, USA). In this study, deionized water produced by the Milli-Q^®^ (Millipore, Bedford, MA, USA) water purification system was used.

### 3.2. Quantitative Determination of 10-HDA in RJ and Formulations

The 10-HDA content in samples was analyzed with the HPLC method using a Waters 2695 chromatographic system, with diode matrix detector Waters 996. The chromatographic conditions were two eluents: trifluoroacetic acid and acetonitrile. The columns were ACE C18 (250 × 4, 6 mm), the volume of injection was 10 µL, the speed of injection was 1 mL/min, the column temperature was 25 °C, and the wavelength for detection was 210 nm. The concentrations were determined and summarized using the Empower 3 chromatographic Software (Waters Corporation, Milford, CT, USA).

### 3.3. Determination of Antioxidant Activity

#### 3.3.1. DPPH Method

DPPH solution in 96.3% *v/v* ethanol (3 mL, 6 × 10^−5^ M) was mixed with 10 μL of RJ and 10-HDA samples. A decrease in absorbance was determined at λ = 515 nm in a double beam UV/VIS spectrophotometer M550 [57]. This spectrophotometer was used for all antioxidant experiments. Results are expressed as a percentage of the DPPH radical binding of samples.

#### 3.3.2. ABTS Method

First, 3 mL of ABTS⋅+ solution was mixed with 10 μL of samples. A decrease in absorbance was measured at λ = 734 nm [58].

#### 3.3.3. FRAP Assay

The FRAP solution included TPTZ (0.01 M dissolved in 0.04 M HCl), FeCl_3_ × 6H_2_O (0.02 M in water), and acetate buffer (0.3 M, pH 3.6) (ratio 1:1:10). First, 3 mL of a freshly prepared FRAP reagent was mixed with 10 μL of all royal jelly and 10-HDA samples. An increase in absorbance was recorded at λ = 593 nm [59].

### 3.4. Determination of Antimicrobial Activity

The bactericidal properties of RJ and 10-HDA were evaluated in vitro using the agar diffusion method described in previous works [60]. Briefly, the method using the Müller–Hinton agar (Mueller–Hinton agar Oxoid LTD (CM 0337), Basingstoke, UK) was used. In vitro studies were performed with the Gram-positive and Gram-negative reference bacteria strains Staphylococcus aureus (ATCC 25923), *Escherichia coli* (ATCC 25922), *Bacillus cereus* (ATCC 10987), *Pseudomonas aeruginosa* (ATCC 27853), and yeast *Candida albicans* (ATCC 10231). Fluid Mueller–Hinton agar was prepared and poured into 10 cm diameter Petri dishes, 35 mL each, and left in the horizontal position to thicken. Bacterial and yeast strains were spread on the surface of the thickened medium, and six wells (7 mm diameter) were made in each Petri dish and filled with 0.1 mL of RJ and 10-HDA samples. The plates were incubated for 24 h at 36 °C. The antibacterial activity was evaluated after 24 h cultivation by measuring the diameter of the transparent areas around the wells. No transparent area was interpreted as negative antimicrobial activity. Each experiment was carried out in triplicate.

### 3.5. Preparation of Thermosensitive In Situ Gels Containing Royal Jelly

The thermosensitive gels were prepared according to the properties of the materials used. First, benzalkonium chloride was dispersed in part of water. Then, an appropriate amount of poloxamer P407 was weighed and slowly dispersed in cold sterile water for injections under continuous mixing using a magnetic stirrer (IKAMAG C-MAG HS7 (IKA-Werke GmbH & Co.KG, Staufen, Germany). Sterile P188 10% solution was added while mixing the dispersion; after that, benzalkonium chloride solution was added. The mixture was left in the refrigerator at 4 ± 1 °C until a clear solution was obtained. Then, the prepared solution was autoclaved and then cooled down and kept in the refrigerator for 24 h to equilibrate. Then, the solutions were inspected for any visual particles and filtered as needed.

The RJ and 10-HDA solutions were prepared in the part of sterile water in aseptic conditions (using Esco Airstream laminar). The appropriate amounts were weighed and mixed with sterile water and then filtered through a 0.45 µm sterile filter and then added to the prepared polymer solution and stirred until they were completely homogenous and clear. The final product was filtered through a sterile filter, and formulations were stored in glass vials at 4 ± 1 °C for further use.

### 3.6. The Determination of Physicochemical Properties of In Situ Gels

The pH of the prepared in situ gels was determined using a pH meter at 25 ± 1 °C, which is used for determining the pH of liquid form (pH meter 766 with Knick SE 100N electrode). The pH meter was calibrated with standard buffer solutions at pH 4.0 and 7.0 and recalibrated for each new sample.

The refractive index of in situ gels was evaluated at 25 ± 2 °C with a refractometer (Metler) according to the manual provided with the instrument.

The dynamic viscosity of in situ gels was determined using a Vibro Viscometer SV-10 (A&D Company, Ltd., Tokyo, Japan). The studied substance was placed into a special container for measurement. Subsequently, the container was fixed on the working surface of the device, and sensors were submerged into the studied gels. The rotation speed of a cylindrical spindle was 10.0 rev/min. The length of every measurement was 10 seconds. The viscosity was assessed at 4, 22, and 35 °C.

Rheological determination of the sol–gel temperature was performed using a MCR102 modular compact rheometer (Anton Paar, Graz, Austria) with a cone and plate fixture with 2° cone angle. Briefly, the samples of in situ gels were carefully applied to the lower plate of the rheometer, ensuring that the formulation was spread evenly, and it was allowed to equilibrate at least 5 min prior to the analysis.

A temperature sweep analysis was performed at a fixed frequency of 0.1 Hz over the temperature range of 4–40 °C, with the temperature being increased at 1 °C/min. Then, the storage modulus (*G*′) and loss modulus (*G*″) were determined, *T*_sol/gel_ was considered to be the temperature at which both of the moduli changed rapidly, as proposed in the literature [61,62]. The analyses were performed in triplicate.

### 3.7. The Determination of Antioxidant Activity of Prepared In Situ Gels Using DPPH Method

DPPH solution in 96.3% *v/v* ethanol (3 mL, 6 × 10^−5^ M) was mixed with 10 μL of RJ and 10-HDA samples. A decrease in absorbance was determined at λ = 515 nm in a double beam UV/VIS spectrophotometer M550 [57]. This spectrophotometer was used for all antioxidant experiments. Results are expressed as a percentage of DPPH radical binding of samples.

### 3.8. Release Studies from In Situ Gel Formulations

The release studies measuring the amount of 10-HDA released from the formulations were measured through a dialysis membrane using modified Franz diffusions cells. The receptor compartment contained freshly prepared artificial tear fluid (NaCl 0.67 g, NaHCO_3_ 0.20 g, CaCl_2_ 2H_2_O 0.008 g) and distilled deionized water up to 100 g [63], and the donor compartment contained 1 mL of in situ gel. A dialysis tubing cellulose membrane (Sigma Aldrich, St. Louis, MI, USA) was placed between the donor and receptor compartments. The receptor medium was stirred using the hotplate magnetic stirrer IKAMAG C-MAG HS7 (IKA-Werke GmbH & Co.KG, Staufen, Germany). The temperature throughout the whole experiment was maintained at 37 ± 1 °C. Samples of 1 mL were taken at predetermined regular intervals and replaced with the same volume of artificial tear fluid. The samples were analyzed by the developed and validated HPLC method.

### 3.9. Stability Study of In Situ Gel Formulations

The stability of the in situ gels containing RJ and 10-HDA was evaluated after 1 and 2 weeks. The formulations were kept in the fridge (temperature 4–8 °C). The stability parameters measured were pH and the amount of 10-HDA. The formulations were also inspected for any visible particles.

The pH was evaluated using a pH meter at 25 ± 1 °C, which is used for determining the pH of liquid form (pH meter 766 with Knick SE 100N electrode). The pH meter was calibrated with standard buffer solutions at pH 4.0 and 7.0 and recalibrated for each new sample.

The amount of 10-HDA in the formulations was evaluated by the developed and validated HPLC method, which is described above.

### 3.10. SIRC Cell Viability Using Mtt Dye

Cell viability studies using the rabbit corneal epithelial cell line SIRC (from the American Type Culture Collection; ATCC) were performed using MTT method [64]. Briefly, cells were cultivated according to the protocol provided by the ATCC, using Eagle‘s minimum essential medium with 10% fetal bovine serum and 1% antibiotic solution (penicillin/streptomycin solution stabilized with 10,000 U penicillin and 10 mg streptomycin/mL) to avoid the growth of unwanted bacteria. Flasks (75 cm^2^) were kept in the incubator at 37 °C with a minimum relative air humidity of 95% in an atmosphere of 5% CO_2_. The MTT method reveals the number of cells with metabolically active mitochondria; living cells with active mitochondria metabolize MTT dye (tetrazolium bromide salt) to formazan, which is purple and can be identified spectrophotometrically. The absorbance was measured at 570 nm.

SIRC cells were seeded in a 96-well plate (1 × 10^4 ^ cells/well). After reaching 50–60% of confluence, the cultures were incubated with various amounts of in situ gel formulations and RJ as well as 10-HDA solutions for 24 h. The control cells were incubated with culture medium only without the formulations tested. After the 24 h treatment, the medium was removed, and the cells were washed twice with 100 µL of pH 7.4 PBS. The PBS was removed, and 100 µL of a mixture of PBS and MTT (9:1) were added to each well and covered. The cells were incubated for 3 h, and the solution was removed, leaving the formazan crystals at the bottoms of the wells. To solubilize the crystals, 100 µL of dimethyl sulfoxide was added to each well, and the level of absorbance was read with the Tecan plate reader. Each experiment was carried out in triplicate. The cell viability was calculated with Equation (1):

(1) $$Cell\;viability\;(\%)=\frac{ABS_{s}}{ABS_{control}}\times 100$$

where *ABS_s_* is the absorbance of cells treated with test formulations and *ABS_control_* is the absorbance of control cells. The results were expressed as the percentage of cells alive (cell viability).

### 3.11. In Vitro Eye Irritation Test Using SIRC Cell Culture Line

The in vitro eye irritation test using the SIRC cell culture line was performed in order to screen the potential of in situ gels for being irritant prior to the in vivo test to avoid the use of animals. The protocol used for the test was introduced by Takahashi et al. [65]. Briefly, SIRC cells were seeded in 96-well plates with a density of 5.0 × 10^3^ cells/well. After incubation for 48 h, the cells reached full confluence and were exposed to 100 μL of the in situ gel formulations or RJ and 10-HDA solutions for 30 min. After exposure, the cells were washed with PBS, and cell viability was assessed using MTT assay. The results were expressed as the percentage of cells alive. Each experiment was carried out in triplicate.

### 3.12. Statistical Analysis

Results are presented as means ± standard deviation of 3–5 experiments carried out. Statistical analysis of experimental data was performed using SPSS software (version 27.0) (IBM statistics, Chicago, IL, USA) and Microsoft Office Excel 2016 (Microsoft, Redmond, WA, USA) computer software. One-way ANOVA (Turkey’s honestly significant difference criteria) was used for statistical analysis. Spearman’s rank coefficient was used for correlation analysis. A value of *p* < 0.05 was taken as the level of significance.

## 4. Conclusions

The results of the antioxidant and antimicrobial activity assessment have shown that Lithuanian RJ possesses higher antioxidant activity than pure 10-HDA, and the antimicrobial assay by diffusion into agar showed that both RJ and 10-HDA exhibit strong antimicrobial activity against the reference bacterial and yeast cultures.

The in situ gels prepared with Lithuanian RJ and pure 10-HDA possessed the physicochemical properties that have met suitable requirements for ophthalmic preparations.

The cell viability tests using SIRC-cultured cells confirmed that the formulations prepared were non-toxic, and the prepared in situ gels were suitable for further evaluation in vitro and in vivo.

## Figures and Tables

**Table 1:** The amount of 10-HDA in RJ samples determined by HPLC.

RJ Samples	The Amount of 10-HDA (%, *w/w*)
RJ1	2.58 ± 0.13
RJ2	3.06 ± 0.16
RJ3	3.63 ± 0.19

**Table 2:** Antioxidant activity of RJ and 10-HDA assessed by ABTS, DPPH, and FRAP assays.

Samples	ABTS (%)	DPPH (%)	FRAP (%)
5% (*w/v*) RJ suspension in PBS	51.563 ± 2.689	48.277 ± 3.189	45.473 ± 2.285
1% (*w/v*) RJ suspension in PBS	19.385 ± 1.014	18.502 ± 0.956	15.054 ± 0.853
0.1% (*w/v*) 10-HDA	8.593 ± 0.627	9.851 ± 0.568	0.936 ± 0.039
0.2% (*w/v*) 10-HDA	8.645 ± 0.572	8.465 ± 0.563	0.941 ± 0.045
0.5% (*w/v*) 10-HDA	8.128 ± 0.517	8.567 ± 0.496	0.881 ± 0.041
1% (*w/v*) 10-HDA	8.373 ± 0.419	8.333 ± 0.472	0.818 ± 0.037

**Table 3:** Antimicrobial activity of RJ and 10-HDA.

Bacterial Strain	RJ Samples (mm disc)	1% (*w/v*) 10-HDA (mm disc)
*Pseudomonas aeruginosa*	13.667 ± 1.33	14.264 ± 0.71
*Candida albicans*	13.33 ± 0.67	25.164 ± 1.26
*Escherichia coli*	15.27 ± 1.09	18.334 ± 0.93
*Bacillus cereus*	8.333 ± 0.42	3.331 ± 0.16
*Staphilococcus aureus*	11.333 ± 1.03	15.141 ± 0.78

**Table 4:** Compositions of the in situ forming gels with RJ and 10-HDA.

GC ^1^	RJ (% *w/w*)	10-HDA (% *w/w*)	P407 (%*w/w*)	P188 (10% Solution *w/w*)	Benzalkonium Chloride (% *w/w*)	Sterile Water (%, *w/w*)
N1	0.5	-	13	15	0.004	71.496
N2	0.75	-	13	15	0.004	71.246
N3	1	-	13	15	0.004	70.996
N4	0.5	-	15	13	0.004	71.496
N5	0.75	-	15	13	0.004	71.246
N6	1	-	15	13	0.004	70.996
N7	0.5	-	18	10	0.004	71.496
N8	0.75	-	18	10	0.004	71.246
N9	1	-	18	10	0.004	70.996
N10	-	0.001	13	15	0.004	71.976
N11	-	0.0015	15	13	0.004	71.976
N12	-	0.002	18	10	0.004	71.976

^1^ GC—gel composition.

**Table 5:** Physicochemical properties of the prepared in situ gels. Data represent mean standard deviation (SD), *n* = 3.

GC	Visual Appearance at 4 °C	RI (at 37 °C)	pH	$T_{Sol-Gel}$ (±1 °C)	Dynamic Viscosity (mPa·s)		
					4 °C	22 °C	35 °C
N1	Clear, transparent liquid	1.414 ± 0.07	5.7 ± 0.29	29	18.0 ± 0.9	22.0 ± 2.1	53.5 ± 1.8
N2		1.392 ± 0.06	5.6 ± 0.28	29.5	17.1 ± 0.8	18.1 ± 0.9	52.6 ± 2.1
N3		1.351 ± 0.07	5.96 ± 0.31	30.5	18.2 ± 1.2	21.4 ± 1.3	51.9 ± 1.7
N4		1.373 ± 0.08	5.29 ± 0.26	27.5	21.5 ± 2.1	20.7 ± 1.6	68.2 ± 2.3
N5		1.392 ± 0.08	5.15 ± 0.25	28	19.5 ± 1.4	22.7 ± 1.8	75.9 ± 2.6
N6		1.424 ± 0.08	5.24 ± 0.27	27	21.2 ± 1.9	25.1 ± 2.3	78.4 ± 2.2
N7		1.383 ± 0.07	5.87 ± 0.32	24.5	34.2 ± 2.5	82.3 ± 3.1	8240 ± 11.1
N8		1.432 ± 0.09	5.57 ± 0.23	25	32.5 ± 2.7	77.8 ± 2.8	7920 ± 9.8
N9		1.322 ± 0.06	5.95 ± 0.35	26.5	43.2 ± 3.1	79.5 ± 3.7	8430 ± 10.6
N10		1.362 ± 0.06	5.87 ± 0.23	30	16.9 ± 0.8	19.4 ± 1.1	44.1 ± 2.1
N11		1.410 ± 0.07	5.23 ± 0.29	27	17.5 ± 1.1	20.2 ± 1.6	67.2 ± 3.4
N12		1.391 ± 0.07	4.98 ± 0.17	25.5	19.1 ± 1.6	78.3 ± 3.7	>10000

GC—gel composition, RI—refractive index.

**Table 6:** The amount of 10-HDA in all formulations determined by HPLC. Data represent the mean standard deviation (SD), *n* = 3.

Formulation	The Amount of 10-HDA in Sample (µg/mL)
N1	19.254 ± 2.524
N2	34.218 ± 2.915
N3	48.172 ± 4.376
N4	16.634 ± 2.214
N5	29.656 ± 3.054
N6	41.563 ± 2.321
N7	18.679 ± 0.923
N8	32.641 ± 1.732
N9	44.674 ± 2.364
N10	16.563 ± 0.928
N11	38.674 ± 1.834
N12	48.565 ± 2.329

**Table 7:** The results of the stability of the in situ gel formulations evaluated by pH and the amount of 10-HDA in samples after two weeks. Data represent mean standard deviation (SD), *n* = 3.

Formulation	After Preparation		After 1 Week		After 2 Weeks	
	pH	10-HAD (µg/mL)	pH	10-HDA (µg/mL)	pH	10-HDA (µg/mL)
N1	5.7 ± 0.29	19.254 ± 2.524	5.61 ± 0.15	19.026 ± 2.282	5.46 ± 0.14	18.698 ± 2.285
N2	5.6 ± 0.28	34.218 ± 2.915	5.43 ± 0.23	34.205 ± 2.254	5.34 ± 0.16	33.832 ± 3.125
N3	5.96 ± 0.31	48.172 ± 4.376	5.84 ± 0.18	48.026 ± 3.563	5.47 ± 0.21	47.785 ± 4.018
N4	5.29 ± 0.26	16.634 ± 2.214	5.18 ± 0.21	16.462 ± 2.452	4.73 ± 0.17	16.427 ± 1.576
N5	5.15 ± 0.25	29.656 ± 3.054	5.09 ± 0.19	29.612 ± 3.835	4.59 ± 0.27	28.986 ± 2.867
N6	5.24 ± 0.27	41.563 ± 2.321	5.16 ± 0.30	41.482 ± 2.259	4.65 ± 0.19	41.340 ± 2.768
N7	5.87 ± 0.32	18.679 ± 0.923	5.82 ± 0.24	18.275 ± 1.037	5.28 ± 0.21	18.461 ± 1.256
N8	5.57 ± 0.23	32.641 ± 1.732	5.46 ± 0.20	32.252 ± 1.626	5.14 ± 0.22	32.603 ± 2.729
N9	5.95 ± 0.35	44.674 ± 2.364	5.78 ± 0.17	44.835 ± 2.645	5.28 ± 0.30	44.220 ±3.155
N10	5.87 ± 0.23	16.563 ± 0.928	5.51 ± 0.25	16.528 ± 1.852	5.03 ± 0.27	16.365 ± 1.564
N11	5.23 ± 0.29	38.674 ± 1.834	5.14 ± 0.27	38.674 ± 2.467	4.83 ± 0.25	38.620 ± 1.936
N12	4.98 ± 0.17	48.565 ± 2.329	4.85 ± 0.26	48.651 ± 3.189	4.76 ± 0.23	47.903±3.462

## Data Availability

All datasets generated for this study are included in the article.

## Sample Availability

The samples for this paper’s experiment are available from the authors.
